# miR-133b down-regulates ABCC1 and enhances the sensitivity of CRC to anti-tumor drugs

**DOI:** 10.18632/oncotarget.17677

**Published:** 2017-05-08

**Authors:** Miao Chen, Daojiang Li, Ni Gong, Hao Wu, Chen Su, Canbin Xie, Hong Xiang, Changwei Lin, Xiaorong Li

**Affiliations:** ^1^ Department of Gastrointestinal Surgery, The Third Xiangya Hospital of Central South University, Changsha, Hunan 410013, P. R. China; ^2^ Laboratory Medical Center, The Third Xiangya Hospital of Central South University, Changsha, Hunan 410013, P. R. China

**Keywords:** colorectal cancer, miR-133b, ABCC1, multidrug resistance, chemosensitivity

## Abstract

Multidrug resistance (MDR) is the main cause of failed chemotherapy treatments. Therefore, preventing MDR is pivotal in treating colorectal cancer (CRC). In a previous study miR-133b was shown to be a tumor suppressor. Additionally, in CRC cells transfected with miR-133b, ATP-binding cassette (ABC) subfamily C member 1(ABCC1) was shown to be significantly down regulated. Whether miR-133b also enhances the chemosensitivity of drugs used to treat CRC by targeting ABCC1 is still unclear. Here, we utilized flow cytometry and high-performance liquid chromatography (HPLC) analysis to identify the ability of miR-133b to reserve MDR in CRC. We then used a dual-luciferase reporter assay to validate that miR-133b targets ABCC1. Further *in vivo* experiments were designed to validate the method in which miR-133b reversed MDR in CRC cells. The results demonstrated that the level of miR-133b was down-regulated and the expression of ABCC1 was up-regulated in drug-resistant CRC cells compared to non-drug-resistant CRC cells. The restoration of miR-133b expression in CRC drug-resistant cells *in vitro* resulted in reduced IC50s to chemotherapeutic drugs, significantly induced G1 accumulation, inhibited growth and promoted necrosis in combination with either 5-fluorouracil (5-FU) or vincristine (VCR), and decreased the expression of ABCC1. The dual-luciferase assay demonstrated that miR-133b directly targets ABCC1. The combination of agomiRNA-133b with chemotherapeutic drugs *in vivo* inhibited tumor growth induced by CRC drug-resistant cells. A xenograft from the *in vivo* model resulted in up-regulated levels of miR-133b and down-regulated levels of ABCC1. Therefore, miR-133b enhances the chemosensitivity of CRC cells to anti-tumor drugs by directly down-regulating ABCC1. This discovery provides a therapeutic strategy in which miR-133b is used as a potential sensitizer for drug-resistant CRC.

## INTRODUCTION

CRC is one of the most commonly diagnosed cancers worldwide with over 1.3 million new cases diagnosed each year [1]. Surgical resection is the primary treatment of CRC, however, chemotherapy is an important adjuvant treatment. 5-FU and VCR are widely used in the treatment of various cancers, and are the standard chief chemotherapeutic drugs for CRC [2, 3]. Patients who are treated with chemotherapeutics rapidly develop resistance to multiple drugs, including structurally and functionally unrelated drugs. This is known as multidrug resistance (MDR) and it is a major obstacle in advanced CRC chemotherapy [4]. Therefore, studies identifying the mechanism in which MDR develops from CRC treatments and studies identifying an effective drug for the reversal of MDR are critical in improving advanced CRC chemotherapy.

MicroRNAs (miRNAs) are a new class of endogenous non-coding RNA molecules. It has been reported that miRNAs are involved in the developmental processes of multiple cancers, including proliferation, migration, and apoptosis by acting as either tumor suppressors or oncogenes [5, 6]. Accumulating evidence reveals that miRNAs can increase the sensitivity of cancer cells to chemotherapeutic drugs. For example, miR-204 can increase the sensitivity of CRC cells to 5-FU, and miR-143 and miR-196 can increase the sensitivity of CRC cells to oxaliplatin [7–9]. MiR-133b, initially considered to be a muscle-specific miRNA, has previously been identified as a tumor suppressor. The expression of miR-133b is down-regulated in colorectal tumors [10, 11]. The role of miR-133b in sensitizing human CRC multidrug-resistant cells to chemotherapeutic drugs remains largely unknown.

ATP-dependent transporters are the most studied and pivotal mechanism of MDR in cancer cells. By using gene arrays in a previous study, we found that the levels of many genes were altered in CRC cells after the transfection of miR-133b. Expression of ABCC1 was reduced more than any other gene in colorectal cancer cells [12]. ABCC1, a member of ATP-binding cassette transporter superfamily, encodes multidrug resistance associated protein 1 (MRP1). MRP1 is an integral membrane glycophosphoprotein with an apparent molecular weight of 190 kDa and functions as a primary active transporter utilizing the energy of ATP binding/hydrolysis [13, 14]. MRP1 was originally isolated based on its elevated expression in the multidrug resistant small cell lung cancer cell line [15]. Subsequent evidence show MRP1 can enhance the MDR of cancer cells, including CRC cells, by transporting a diverse class of amphipathic drug molecules across biological membranes and out of the target cells [16, 17].

In this study, we aimed to address the following questions: is miR-133b responsible for the reversion of MDR in CRC cells and what is its underlying mechanism? What is the relationship of miR-133b and ABCC1? Our results indicated that miR-133b enhances the chemosensitivity of CRC to anti-tumor drugs by directly down-regulating ABCC1, suggesting a potential drug responsible for the reversal of MDR in CRC cells. In short, our results may provide a therapeutic strategy with miR-133b as a potential sensitizer for drug resistant CRC cells.

## RESULTS

### Expression of miR-133b and ABCC1 in CRC multidrug resistant cell lines

**Figure 1:**
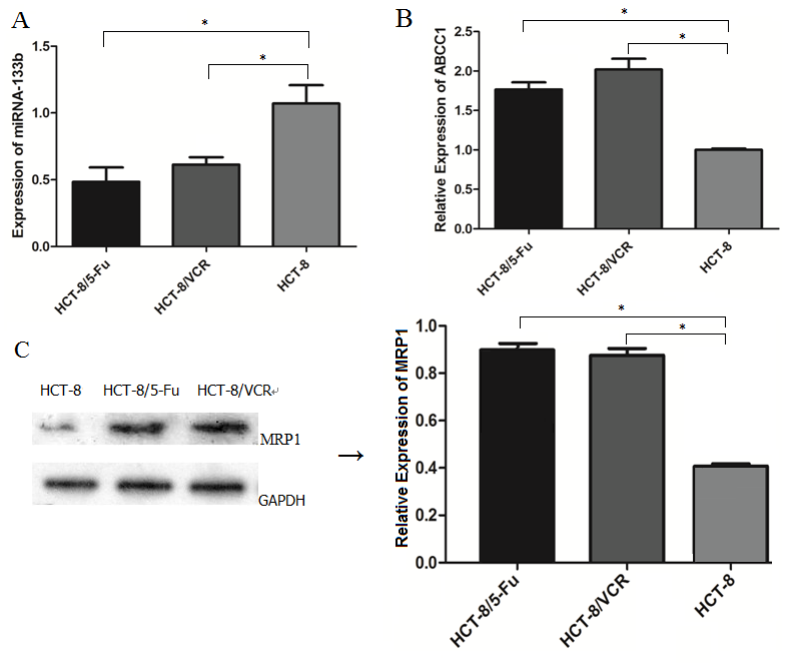
Expression levels of miR-133b and ABCC1 in normal and drug-resistant CRC cell lines **(A)** The expression of miR-133b was decreased in CRC drug-resistant cell lines HCT-8/5-FU and HCT-8/VCR compared with that of normal CRC cell HCT-8, as detected by RT-qPCR. **(B)** Increased expression level of ABCC1 was detected in HCT-8/5-FU and HCT-8/VCR compared with that of HCT-8, as detected by RT-qPCR. **(C)** The expression level of ABCC1 was detected by Western blot analysis, and was increased in HCT-8/5-FU and HCT-8/VCR. The data are expressed as the mean ± standard deviation. The results are representative of three independent experiments. *P<0.05.

To determine the potential pathological implications of altered expression of miR-133b and ABCC1 relevant to MDR in CRC cells, the expression levels of miR-133b and ABCC1 in multidrug resistant cell lines were investigated using RT-qPCR and western blot. As shown in Figure 1A, the expression levels of miR-133b were significantly lower in HCT-8/5-FU and HCT-8/VCR cell lines than the normal CRC cell line, HCT-8. Additionally, the mRNA and protein levels of ABCC1 were significantly higher in HCT-8/5-FU and HCT-8/VCR cell lines compared to the normal CRC cell line, HCT-8 (Figure 1B and 1C). These results suggested that miR-133b and ABCC1 are associated with MDR in CRC cells.

### Combination of miR-133b and either 5-FU or VCR decreased the IC50 of 5-FU and VCR

**Figure 2:**
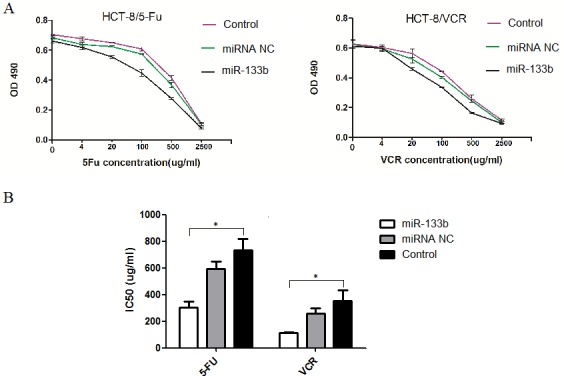
Cell activity assay **(A)** MTT OD values. **(B)** The altered IC50 of 5-FU and VCR in HCT-8/5-FU and HCT-8/VCR cell lines, respectively. The result showed the IC50s of 5-FU and VCR in corresponding drug-resistant cells HCT-8/5-FU and HCT-8/VCR were significantly decreased after transfected by miR-133b. The data are expressed as the mean ± standard deviation. The results are representative of three independent experiments. *P<0.05.

To assess whether miR-133b amplifies the ability of 5-FU and VCR to suppress tumors in CRC multidrug resistant cells, an MTT assay was performed to measure the altered IC50 of 5-FU in HCT-8/5-FU and VCR in HCT-8/VCR cell lines after transfected with miR-133b. The cell activity of HCT-8/5-FU and HCT-8/VCR was significantly inhibited with an increased concentration of 5-FU or VCR in the miR-133b group (Figure 2A). Additionally, the IC50 of 5-FU and VCR were significantly lower compared to the miRNA NC and control groups (Figure 2B). Both results demonstrated that miR-133b could increase the efficacy of 5-FU and VCR on CRC multidrug resistant cells.

### Combination of miR-133b and either 5-FU or VCR inhibited multidrug resistant cell growth

**Figure 3:**
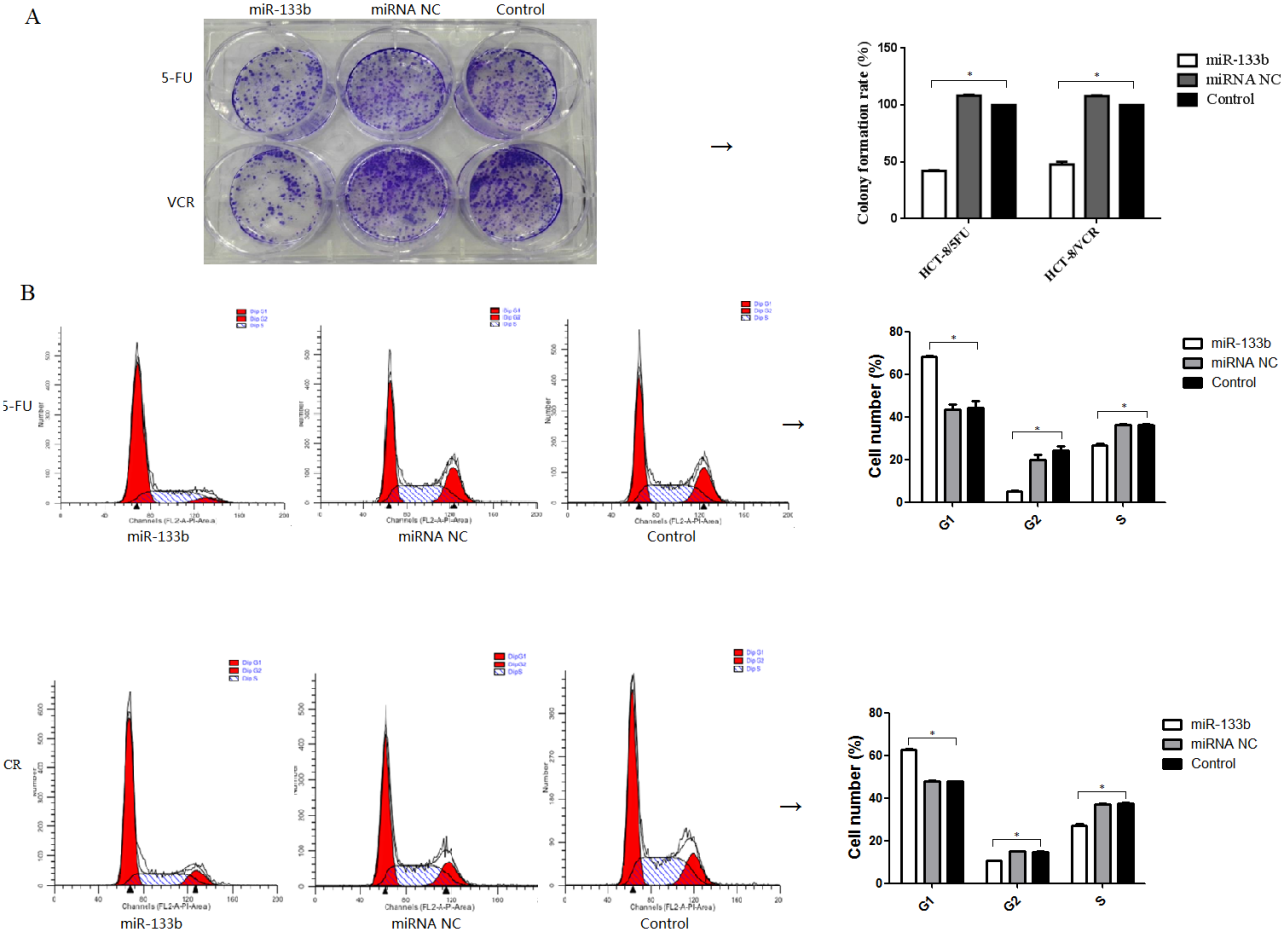
Combination of miR-133b and 5-FU or VCR inhibited multidrug resistant cell growth **(A)** The inhibition of the clone formation ability of HCT-8/5-FU and HCT-8/VCR cells. **(B)** The effect on the cell cycle of HCT-8/5-FU and HCT-8/VCR cells. The percentage of cells in the G1, G2 and S phases significantly altered following transfection with miR-133b. *P<0.05.

To evaluate whether miR-133b enhances the ability of 5-FU and VCR to inhibit the growth of CRC multidrug resistant cell lines, clone formation assays and cell cycle analyses were performed. After treatment with either 5-FU or VCR, the number of colonies formed by HCT-8/5-FU-miR-133b cells and HCT-8/VCR-miR-133b cells were significantly lower compared to both HCT-8/5-FU-miRNA NC cells and HCT-8/VCR-miRNA NC cells, respectively as well as HCT-8/5-FU cells and HCT-8/VCR cells respectively. Statistical analysis revealed that the colony formation rate of HCT-8/5-FU-miR-133b cells and HCT-8/VCR-miR-133b cells was significantly lower than the rate of HCT-8/5-FU-miRNA NC cells and HCT-8/VCR-miRNA NC cells respectively (p<0.05, Figure 3A). After performing cell cycle analysis, it was found that there was significant accumulation of cells in the G1 phase and decreased cell population in the G2 and S phases of the CRC multidrug resistant cell lines after treatment with 5-FU or VCR compared with the miRNA NC and control groups (Figure 3B). This confirms the ability of miR-133b to sensitize CRC multidrug resistant cell lines to chemotherapeutic drugs. These results indicate that miR-133b can enhance the inhibitory effect of 5-FU and VCR on CRC multidrug resistant cell growth.

### The effect of combining miR-133b and either 5-FU or VCR on the rate of cell apoptosis and necrosis levels in multidrug resistant cell lines

**Figure 4:**
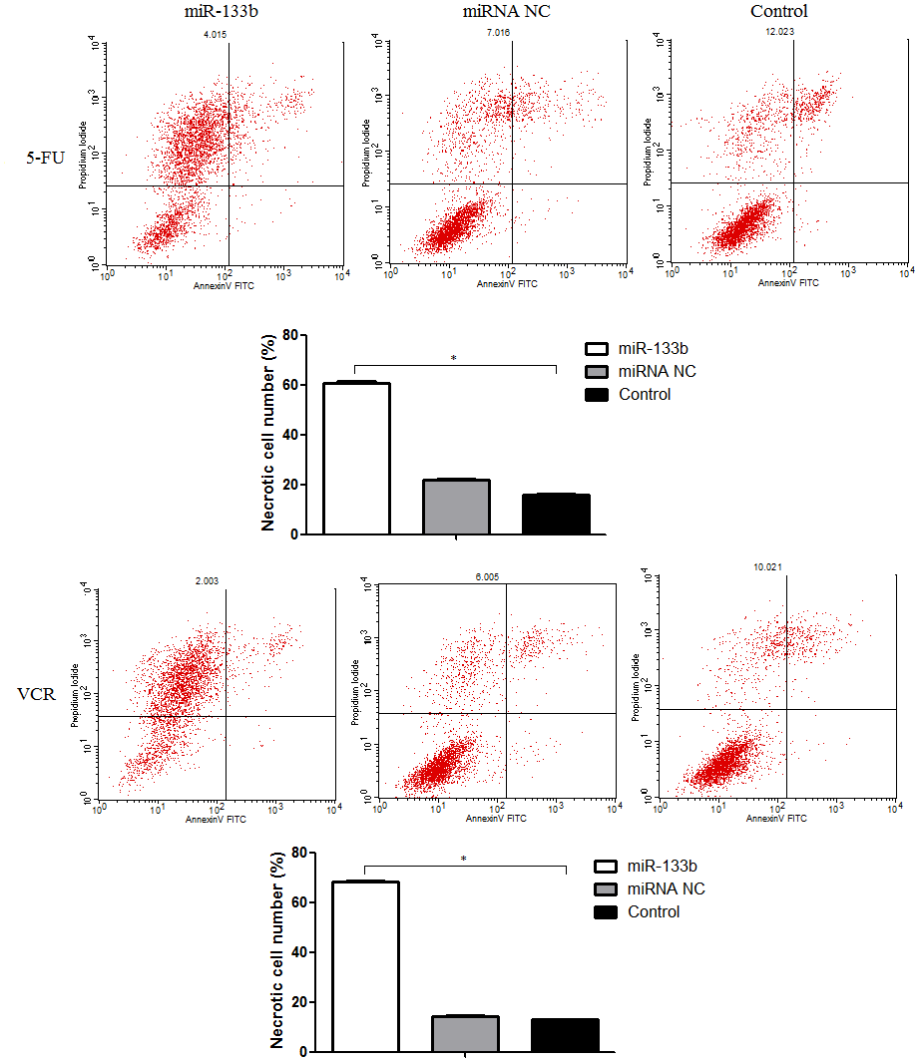
Combination of miR-133b and 5-FU or VCR induced multidrug resistant cell lines necrosis The flow cytometric result is divided into four quadrants by lines. The upper left quadrant (Annexin V-FITC^−^/PI^+^ staining) is necrotic cells; the upper right quadrant (Annexin V-FITC^+^/PI^+^ staining) a late apoptotic cell; the left lower quadrant (Annexin V-FITC^−^/PI^−^ staining) is the normal activity of cells; the lower right quadrant (Annexin V-FITC^+^/PI^−^ staining) are early apoptotic cells. Thus, the rate of necrotic cells significantly increased in miR-133b group. The data are presented as the mean ± standard deviation. The results are representative of three independent experiments. *P<0.05.

To evaluate the ability of miR-133b to decrease cell proliferation in CRC multidrug resistant cell lines, we quantified the number of apoptotic and necrotic cells using flow cytometry (Figure 4). We performed this experiment with the understanding that an increase in cell apoptosis and necrosis levels would indicate a decrease in cell proliferation. After incubation with either 5-FU or VCR, the miR-133b groups had no significant difference in levels of apoptotic cells compared to the miRNA NC and control groups. The rate of cell necrosis, however, was significantly higher in the miR-133b group compared to the miRNA NC and control groups.

### miR-133b represses the expression of ABCC1

**Figure 5:**
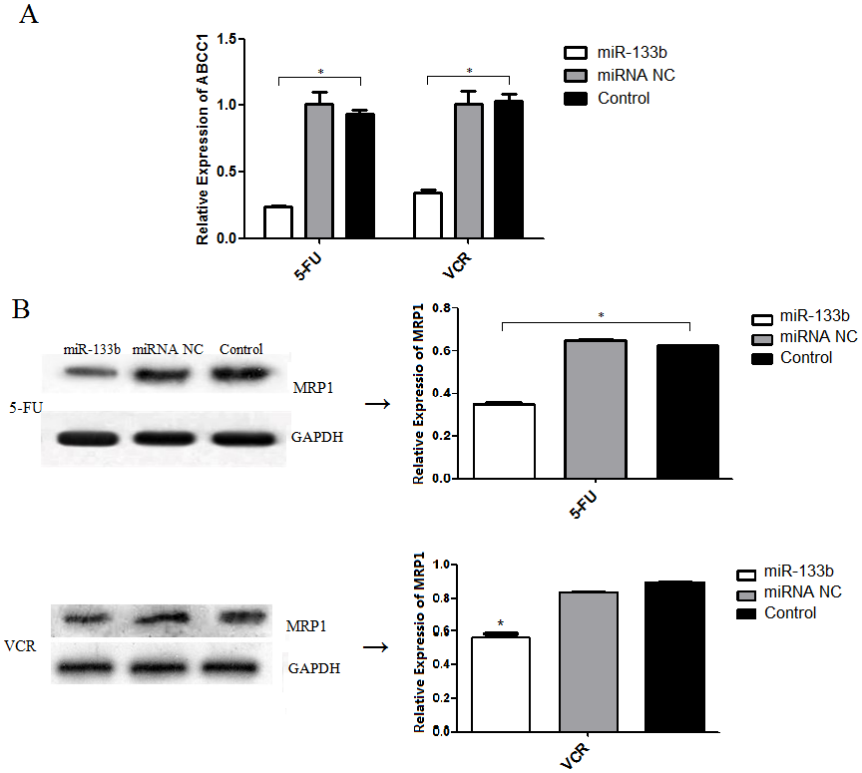
miR-133b inhibits the expression of ABCC1 **(A)** The mRNA level of ABCC1 was detected by RT-qPCR. **(B)** The protein level of ABCC1 was detected by Western blot analysis. The data are presented as the mean ± standard deviation. The results are representative of three independent experiments. *P<0.05.

To investigate the mechanism in which miR-133b decreases MDR, we performed RT-qPCR analysis and a western blot assay. The results showed that both HCT-8/5-FU-miR-133b cells and HCT-8/VCR-miR-133b cells significantly decreased expression of ABCC1 mRNA and protein compared to the cells transfected with miRNA NC and control cells (Figure 5). This suggests that miR-133b decreases MDR by repressing the expression levels of ABCC1 in CRC cells.

### miR-133b targets ABCC1

**Figure 6:**
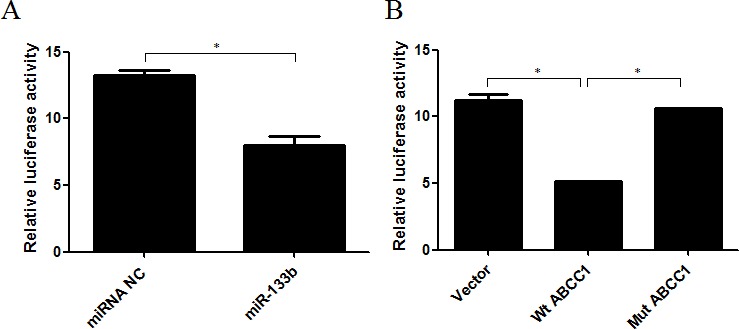
miR-133b directly targets ABCC1 **(A)** The repression of luciferase activity by the 3′UTR of ABCC1 is shown in the miR-133b group compared to the miRNA NC group. **(B)** The mutated 3′UTR of ABCC1 abrogated the repression of luciferase activity mediated by miR-133b. Wt ABCC1: wild type ABCC1; Mut ABCC1: mutant ABCC1. The results are representative of three independent experiments. *P<0.05.

To determine whether miR-133b directly targets ABCC1 in cells, we performed a dual-luciferase assay. This was done by taking a pYr-MirTarget-ABCC1-3U vector containing a luciferase reporter gene whose 3′UTR was inserted with the 3′UTR of ABCC1. Either hsa-miR-133b mimics or miRNA NC were co-transfected into 293 cells. The results showed that miR-133b significantly inhibited the luciferase activity of the reporter gene (Figure 6A). Further, in order to confirm the function of miR-133b directly targeting ABCC1 in cells, a mutant version of the 3′-UTR of ABCC1 in which the predicted miR-133b binding sites were mutated, was constructed and co-transfected with miR-133b into 293 cells. The resulting luciferase activity of the reporter gene of the mutant-ABCC1 group was equivalent to the levels of that in the control group. The luciferase activity in both the mutant-ABCC1 group and control group were significantly different from the wild type-ABCC1 group (Figure 6B). These results confirmed that miR-133b directly targets ABCC1.

### miR-133b enhances the uptake of 5-FU and VCR

**Figure 7:**
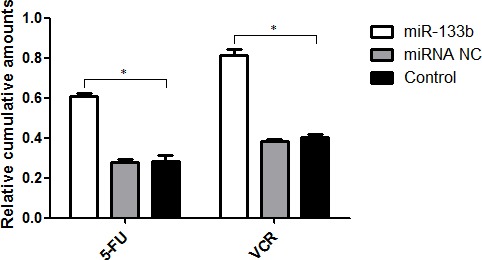
The uptake of 5-FU and VCR in multidrug resistant cell was increased by miR-133b The cumulative amounts of 5-FU or VCR in cells from miR-133b, miRNA NC and control groups were measured. The data are presented as the mean ± standard deviation. The results are representative of three independent experiments. *P<0.05.

To investigate the underlying mechanism in which miR-133b reverses MDR in cells, *in vitro* intracellular uptake of 5-FU and VCR was observed. After incubation with either 5-FU or VCR, the cumulative amounts of 5-FU in VCR in cells of the miR-133b group were significantly higher than that in the miRNA NC and control groups (Figure 7). This indicates that transfected miR-133b effectively increased the uptake of 5-FU and VCR into cells.

### Validated results *in vivo*

**Figure 8:**
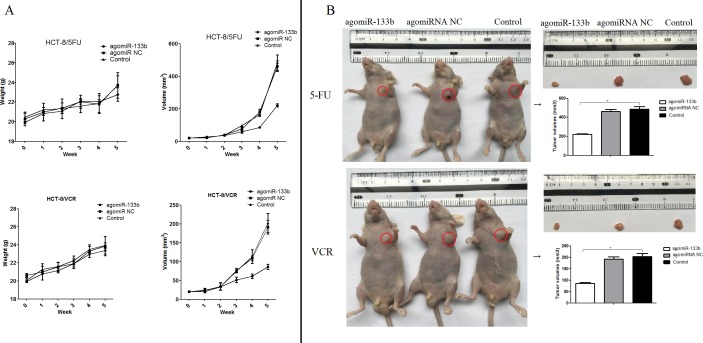
The effect on the growth of xenograft **(A)** Growth curve. The nude mice were weighed and the tumor volume was measured each week. **(B)** Tumor volume. The nude mice were photographed before xenograft tumors were removed. The volume of the xenograft tissues was then measured. The data are presented as the mean ± standard deviation. The results are representative of three independent experiments. *P<0.05.

**Figure 9:**
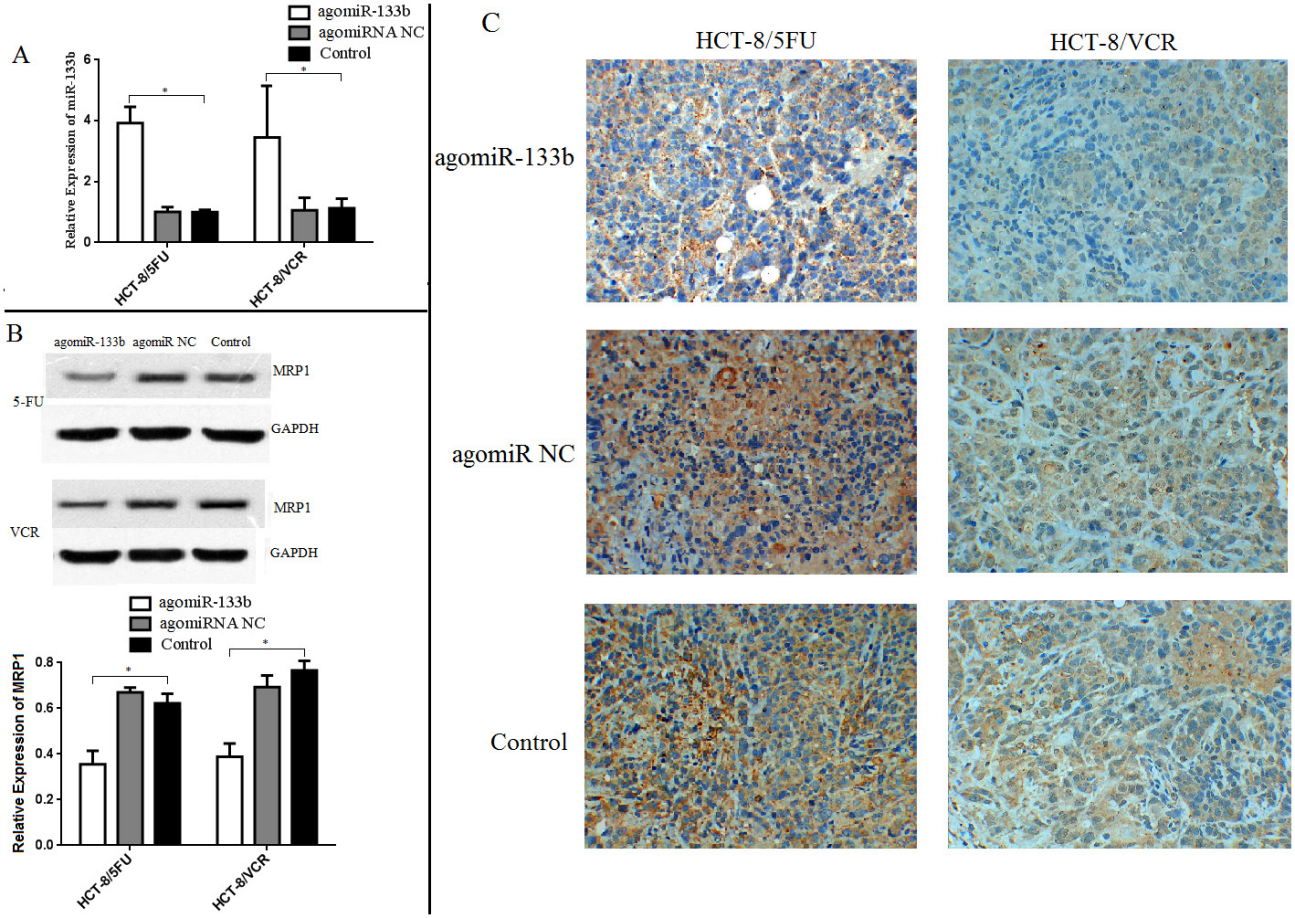
Expression levels of miR-133b and ABCC1 in xenograft **(A)** The expression of miR-133b increased in xenograft treated with agomiRNA-133b. **(B)** Contrarily, the expression level of ABCC1 decreased in the agomiRNA-133b group, as detected by Western blot. **(C)** Immunohistochemical staining of ABCC1 in xenograft also showed the decreased level in agomiRNA-133b group. The data are expressed as the mean ± standard deviation. The results are representative of three independent experiments. *P<0.05.

In order to validate the ability of miR-133b to reverse MDR, nude mice were injected with either agomiRNA-133b treated HCT-8/5-FU cells or agomiRNA-133b treated HCT-8/VCR cells. Three weeks after injection, the mice injected with agomiRNA-133b treated 5-FU and agomiRNA-133b treated VCR cells had significantly decreased tumor volumes with similar body weights compared to both agomiRNA-NC treated 5-FU and agomiRNA-NC treated VCR cells respectively, as well as untreated 5-FU and untreated VCR cells respectively (Figure 8). Furthermore, the relative expression of miR-133b in xenografts was increased after treatment with either agomiRNA-133b treated 5-FU cells or agomiRNA-133b treated VCR cells (Figure 9A). Western blot and IHC results both showed a decrease in the relative level of MRP1 in agomiRNA-133b groups (Figure 9B and 9C). These results further identify that miR-133b enhances the chemosensitivity of CRC cells to chemotherapeutic drugs by suppressing ABCC1.

## DISCUSSION

In the present study, we aimed to explore if one function of miR-133b is to reverse MDR in CRC cell and what the underlying mechanism of such a function would be. In order to identify the relevance of miR-133b with CRC multidrug-resistant cells, the variant level of miR-133b between CRC multidrug-resistant cells and normal CRC cells was detected. The resulting expression level of miR-133b was significantly down-regulated in CRC multidrug-resistant HCT-8/5-FU and HCT-8/VCR cell lines compared with parent CRC cell line, HCT-8. Then, miR-133b was transfected into the HCT-8/5-FU and HCT-8/VCR cell lines which were then incubated with either the chemotherapeutic drug5-FU or VCR respectively. MTT method, plate clone formation assay, and flow cytometric analysis were used to evaluate the effect of miR-133b on the MDR of these cells. Interestingly, the results indicated that the combination of miR-133b and either 5-FU or VCR could inhibit the growth of multidrug resistant cells and enhance the necrosis of multidrug resistant cell lines. This indicates that miR-133b enhances the chemosensitivity of CRC cells. Continual evidence shows that apoptosis is not the only pathway of cell death induced by drugs. When the apoptotic machinery is operative, apoptosis seems to precede necrosis. When the apoptosis program is defective, necrosis takes place as an alternative pathway leading to cell death [18, 19]. Our results demonstrated the synergistic ability of miR-133b and either 5-FU or VCR to induced cell death by triggering cellular necrosis (Figure 4). Furthermore, to validate the positive effect of miR-133b on the chemosensitivity of CRC cells, an *in vivo* experiment was designed. The results showed the combination of miRNA-133b and either 5-FU or VCR could more significantly inhibit the growth of xenograft compared to the combination of agomiRNA NC and either 5-FU or VCR, as well as only either 5-FU or VCR (Figure 8). These results indicate that miR-133b serves as a therapeutic target for reversing MDR of CRC cells. This combinational therapy of miR-133b and chemotherapy drugs may be a promising application for treating patients with CRC.

Over the past decade, a number of studies have revealed that miRNAs act as a tumor suppressor or promoter by regulating up to 60% of human protein-coding genes [20]. In a previous study, we found that a total of 103 genes were differentially expressed (72 up-regulated and 31 down-regulated) in miR-133b-transfected CRC cells. ABCC1 was the most significantly down-regulated gene [12], suggesting ABCC1 may be a direct functional target of miR-133b in CRC cells. To verify this hypothesis, a dual-luciferase assay was performed. The co-transfection of miR-133b mimics and wild-type ABCC1 vector significantly reduced the luciferase activity, while the co-transfection of miR-133b mimics and mutant-type ABCC1 vector regained the luciferase activity. In addition, an inverse correlation between the expression levels of miR-133b and ABCC1 was observed in both the CRC xenograft and cell lines. An over-expression of miR-133b correlated in reduced expression of ABCC1 at both the mRNA and protein levels (Figure 5 and 9). These results suggest that miR-133b directly represses ABCC1 expression leading to enhanced CRC cell sensitivity to chemotherapy drugs.

The ATP-binding cassette (ABC) transporters are important transmembrane proteins encoded by a supergene family. They are primary active transporters that bind and hydrolyze ATP to mediate the efflux of a diverse range of substrates across lipid membranes. ABCC1 encodes ABC transporter MRP1, which was identified in a multidrug resistant lung cancer cell line in 1992 [15]. Numerous studies have shown that MDR is a result of increased drug efflux. Resistant cells accomplish this by over-expressing transporters on the resistant cell's surface. The proteins involved in MDR are typically members of the ABC transporters superfamily [21–23]. MRP1 plays an active role in drug efflux which prevents effective treatment of a range of diseases, including clinical depression, cancer and epilepsy [24–28]. The ability of a drug to be taken up into its target cell is known to be both an initial and a direct determining factor of the drug's efficacy, irrelevant of the drug's treatment mechanisms. In the present study, we found that miR-133b not only represses the expression of ABCC1, but also increases the up-take of 5-FU and VCR into CRC cells (Figure 7). This was determined after performing HPLC analysis, which showed that over-expressed miR-133b could significantly increase the up-take of 5-FU and VCR. This explains the mechanism by which miR-133b reverses MDR in CRC cells (Figure 7). It was also found that over-expressed miR-133b can down-regulate the expression of ABCC1 (Figure 5 and 9). Based on these results, we propose that miR-133b reverses MDR by both increasing the up-take of drugs as well as repressing the expression of ABCC1 and thus reducing drug efflux out of the target cell. To validate that miR-133b directly targets ABCC1 in cells, a dual-luciferase assay was designed (Figure 6). The results were consistent with the results from Lin *et al* who testified TAp63 suppressed metastasis via miR-133b in colon cancer cells only using dual-luciferase assay [29]. We had previously discovered that combinational treatment of miRNA-133b and cetuximab increased inhibition of the growth and invasion of CRC cells by down-regulating EGFR compared to treatment with cetuximab alone [11]. Additionally, Chen *et al* had previously discovered that miRNA-133b decreased glutathione S-transferase π expression resulting in increased ovarian cancer cell sensitivity to chemotherapy drugs [30]. The present study is the first to show miRNA-133b enhancing the sensitivity of CRC to anti-tumor drugs by down-regulating ABCC1 expression. Together, these results suggest that miR-133b can help develop more efficient chemotherapy treatment by increasing the sensitivity of cancer cells to existing chemotherapy drugs through selective regulation of different genes.

In conclusion, we identified an important tumor-suppressive miRNA, miR-133b, as a novel sensitizer for the chemotherapy of human CRC. miR-133b plays a key role in repressing CRC development and progression by increasing the sensitivity of CRC cells to two chemotherapeutic drugs (5-FU and VCR). MiR-133b does this by directly down-regulating ABCC1 resulting in increased up-take of 5-FU and VCR into CRC cells. Therefore, this study highlights the important role of miR-133b in reversing MDR of CRC cells and suggests that miR-133b could be used as both a new biomarker for estimating the level of drug resistance in CRC cells and a method to developing more efficient chemotherapy treatments for CRC.

## MATERIALS AND METHODS

### Ethical standard protocol

All animal protocols used in this study have been revised and approved by The Third Xiangya Hospital, Central South University Animal Care and Use Committee (Certification Number: LLSC(LA) 2015-002).

### Cell culture

Human colorectal adenocarcinoma cell line HCT-8, and its drug-resistant cell HCT-8/VCR were obtained from the Cell Center of Xiangya School of Medicine, Central South University (Hunan, China), HCT-8/5-FU cell was purchased from Kaiji company, Nanjing, China. The cells were maintained in RPMI1640 with 10% heat-inactivated FBS at 37°C incubator containing 5% CO_2_.

### Transfection of miR-133b mimics

Hsa-miR-133b oligonucleotide mimics (sequence: Forward, 5′-UUUGGU CCCCUUCAACCAGCU A-3′; Reverse, 5′-GCUGGUUGAAGGGGACCA AAU U-3′), and miRNA NC (sequence: Forward, 5′-UUCUCCGAACGUGUCACG-3′ UTT-3′; Reverse, ACGUGACACGUUCGGAGAATT-3′) were synthesized by Yingrun (Changsha, China) and were cloned into the pYr vector. HCT-8/5-FU and HCT-8/VCR cells were seeded in RPMI1640 media containing serum in 6-well plates and incubated for 24h. The cells were then transfected using Lipofectamine 2000 (Invitrogen, USA) in antibiotic-free Opti-MEM medium with miR-133b mimics or miRNA NC with a final RNA concentration of 50 nM. The following experiments were carried out 24h after transfection.

### Real-time PCR

Total RNA was isolated from HCT-8, HCT-8/5-FU, HCT-8/VCR, transfected HCT-8/5-FU and HCT-8/VCR with miR-133b or miRNA NC cells using RNeasy Mini Kit (Omega, MT USA) in accordance with manufacturer's recommendations. Roughly 500 ng of extracted total RNA was reverse-transcribed into cDNA using the Primer Script RT reagent kit (Takara Bio, Inc., Otsu, Japan). The relative mRNA expression levels of ABCC1 were detected using Real-Time Quantitative PCR SYBR Green detection reagent (Cowin Biotech Co., Ltd., Beijing, China), with specific primers (Forward, 5′-GC CTGTTTTGGTAAAGAACTGGAAG-3′; Reverse, 5′-CC TTGGAACTCTCTTTCGGCTG-3′). Simultaneously, GAPDH ((Forward, 5′-GAAGGTGAAGGTCGGAGT-3′; Reverse, 5′-CATGGGTGGAATCATATTGGAA-3′) was used as a control for normalizing the relative expression of ABCC1. MiRNA reverse transcription was performed using an All-in-One™ miRNA qRT-PCR Detection kit (GeneCopoeia, Inc., Rockville, MD, USA) to measure the relative expression levels of miR-133b. The relative expression of miR-133b was normalized to that of U6-snRNA (Forward, 5′-CTCGCTTCGGCAGCACA-3′; Reserse, 5′-AACGCTTCACGAATTTGCGT-3′) using the 2^−ΔΔCT^-method. The PCR amplification instrument is from Thermo Fisher Scientific Company.

### Western blot analysis

HCT-8, HCT-8/5-FU and HCT-8/VCR, transfected HCT-8/5-FU and HCT-8/VCR with miR-133b or miRNA-NC cells were collected in the phase of logarithmic growth, and were then lysed with lysis buffer. After boiling and measuring the concentration, 50 μg of protein lysates for each sample were loaded onto a 10% resolving polyacrylamide gels (SDS-PAGE), and gel electrophoresis was run for 1 h. Separated proteins were then transferred to polyvinylidenedifluoride (PVDF) membranes by running electrophoresis overnight. After blocking the membranes with blocking buffer for 1h, the membranes were then incubated with MRP1 primary antibodies (Santa Cruz Biotechnology, Inc, USA) overnight at 4°C. After incubation with HRP-anti-IgG secondary antibodies (SanYing Company, China), the signal was visualized by HRP substrate. Finally, the intensity of the bands was measured by AQM Advance 6 software.

### MTT assay

The transfected cells and control cells were seeded into 96-well plates at a density of 1×10^4^ cells/well. The cells were incubated overnight at 37°C with 5% CO_2_. Cells were treated with corresponding chemotherapeutic drugs 5-FU or VCR (Kaiji Company, Nanjing, China) respectively at 0, 4, 20, 100, 500, 2500μg/ml for 24h prepared by medium with 10% FBS. Meanwhile, HCT-8 cells were used for measuring the IC50 of 5-FUand VCR. Then, 10 μl MTT (0.5 mg/ml) was added to each well and incubated for 4h. After removing the supernatant, the reaction was terminated by the addition of 200μl dimethyl sulfoxide (DMSO) and incubating at 37°C for 15min. The absorbency was measured with a Bio-Rad microplate reader (Bio-Rad, Hercules, CA, USA) at a wavelength of 490 nm. Each assay was performed in triplicate.

### Plate clone formation assay

The transfected cells and control cells were seeded in 24-well plates (200 cells/well) and treated with30μg/ml of either 5-FU or VCR (both are chemotherapeutic drugs) 12h later. The cells were then incubated at 37°C with 5% CO_2_ for 15 days. Next, the cells were washed twice with PBS and fixed in 75% methanol for 15min. The cells were then stained with 1% methylrosanilinium chloride. Finally, the visible colonies were counted using an inverted microscope and the relative clone formation ability was calculated as follows: (mean experimental clone number/mean control clone number) ×100%.

### Flow cytometric analysis

The transfected cells were incubated in the 6-well plates for an additional 24h. Therefore, 48h after initial transfection, the cells were then incubated with 30μg/ml 5-FU or VCR for 24h at 37°C with 5% CO_2_. The cells were then harvested and washed with phosphate-buffered saline (PBS). Subsequently, cells were fixed in 70% ethanol at 4°C overnight. The fixed cells were washed with PBS and resuspended in propidium iodide, AnnexinV-FITC (BD Company, USA) for 15min in the dark at room temperature and immediately analyzed by flow cytometry (Beckman Coulter, USA).

### High-performance liquid chromatography (HPLC) analysis

The transfected cells and control cells were seeded in 6-well plates and incubated with 30μg/ml of 5-FU or VCR for 30min. The cells were washed with ice-cold PBS twice, and then disrupted and harvested in cold PBS. The supernatant was obtained by centrifuged at 15,000g for 20min. The protein concentration was assayed using the Bradford method. Then, the uptake of 5-FU or VCR was measured using an Agilent 1200 series HPLC system (Santa Clara, CA, USA). The supernatant was injected into the chromatographic column (20RBAX Eclipse XDB-C18column) at 25°C. The flow rate was 1.0 ml/min and the detection wave length was at 265 nm.

### Dual-luciferase assay

Site-directed mutagenesis of the miR-133b target sites in the ABCC1 3′-UTR (5′-GGTGCCCTGAGACAGACACACAGCCTCACGCCCCCAGGAATGCAAGTGGTTTCCTGGTGCTTCCCACGGAGGAGTTTTGGCAGCCAGACTTCTGGAGGAATTGGTTGTATAGAAGATCCTAGT**GACCAA**ATTCAGCCTACTGCCTCGGATCTCTCCAGCCGAAGTCTGTGGACTGCAAGTCTTTGAGATGCTTCTGGCTCCCATCACCTCTAACATCCTTGTCTGGGTCTACCAGGA-3′) was performed by removing the 6 bases (5′-GACCAA-3′, highlighted above) of the 124-129 base ABCC1 sequence, and pYr-MirTarget-ABCC1-3U-Mut was constructed. The experiments were conducted in two parts. In the first part, cells were co-transfected with pYr-MirTarget-ABCC1-3U and Hsa-miR-133b mimics (50 Nm) as miR-133b group, or miRNA NC (50 nM) as NC group. In the second part, cells were co-transfected with hsa-mir-133b mimics (50 Nm) and pYr-MirTarget as 133b-Vector group, or pYr-MirTarget-ABCC1-3U as 133b-Wt group, or pYr-MirTarget-ABCC1-3U-Mut as 133b-Mut group and then incubated for 48h. Cells were then transfected with pYr-MirTarget Renilla luciferase plasmid and incubated for 48h. Cells were then assayed using the Dual-Luciferase Reporter Assay System (Yingrun, Changsha China). The luciferase activity was normalized for transfection efficiency with co-transfected Renilla. Luminescence was quantified using a luminometer (Agilent, USA). All experiments were performed in triplicate.

### Animal experiment [8, 31]

BALB/c nude mice (n=18; male; 5-6 weeks old; weight, approximately 20g) were obtained from SJA Lab Animal Co., Ltd (Hunan, China. License number: SCXK (Xiang) 2013-0004). The mice were maintained under specific pathogen-free conditions with a humidity of 30-40%, a 12h dark and light cycle and access to food and water. The mice were randomly divided into 5-FU and VCR groups and separately injected with either HCT-8/5-FU or HCT-8/VCR cells. The original treatment was injected into the right flank of mice. Intraperitoneal injections treatments 5-FU or VCR, respectively, were repeated every other day for 28 days. Additionally, each group was further divided into a miR-133b subgroup treated with agomiRNA-133b, a negative control subgroup treated with agomiRNA-NC, and a blank subgroup. This treatment was injected intratumorally at a dose of 1 nmol (diluted in 20μL phosphate-buffered saline) every 4 days until seven injections per mouse had been performed. Each subgroup contained three mice. The mice were fed continuously for 5 weeks. The volume of each xenograft and the weight of each mouse were recorded once a week. The mice were sacrificed and the tumors were resected and measured. Tumor volume was calculated according to the formula: Volume (mm^3^) = width^2^× length/2.

### The profiling of miR-133b and ABCC1 in xenograft

The relative expression of miR-133b in tumor tissues was detected using Real-Time Quantitative PCR method, and the levels of ABCC1 were tested with western blot according to the protocol *in vitro* as above. Furthermore, immunohistochemical technique (IHC) was used to detect MRP1. Xenograft tumors were removed, fixed in 10% phosphate-buffered formalin and embedded in paraffin for sectioning (5 mm) on a rotary microtome. The sections were treated with MRP1 antibody (Santa Company, USA) overnight at 4°C at an optimal working dilution of 1:1000. After sufficient rinses in phosphate buffered saline (PBS), the sections were incubated with secondary antibody (Abcam, USA) linker incubation for 15 minutes. The slide where then stained with 3, 39-diaminobenzidine tetrahydrochloride (DAB) for 10 minutes and counterstained with hematoxylin, dehydrated and mounted.

### Statistical analysis

Statistical analyses were carried out using two-sided Student's *t*-test by SPSS software (version 19.0; IBM SPSS, Armonk, NY, USA), and the results are presented as the mean ± standard deviation (SD). All data were analyzed using GraphPad Prism version 5.01 (GraphPad Software, Inc., La Jolla, CA, USA). IC50 values were calculated using regression analysis. P-values <0.05 were considered statistically significance.
